# Supplementary figures and images for: Effect of Peptides from Plasma of Patients with Coronary Artery Disease on the Vascular Endothelial Cells
Source: Medicina (Kaunas). 2023 Jan 27;59(2):238. doi: 10.3390/medicina59020238 (PMC10003965; doi:10.3390/medicina59020238)

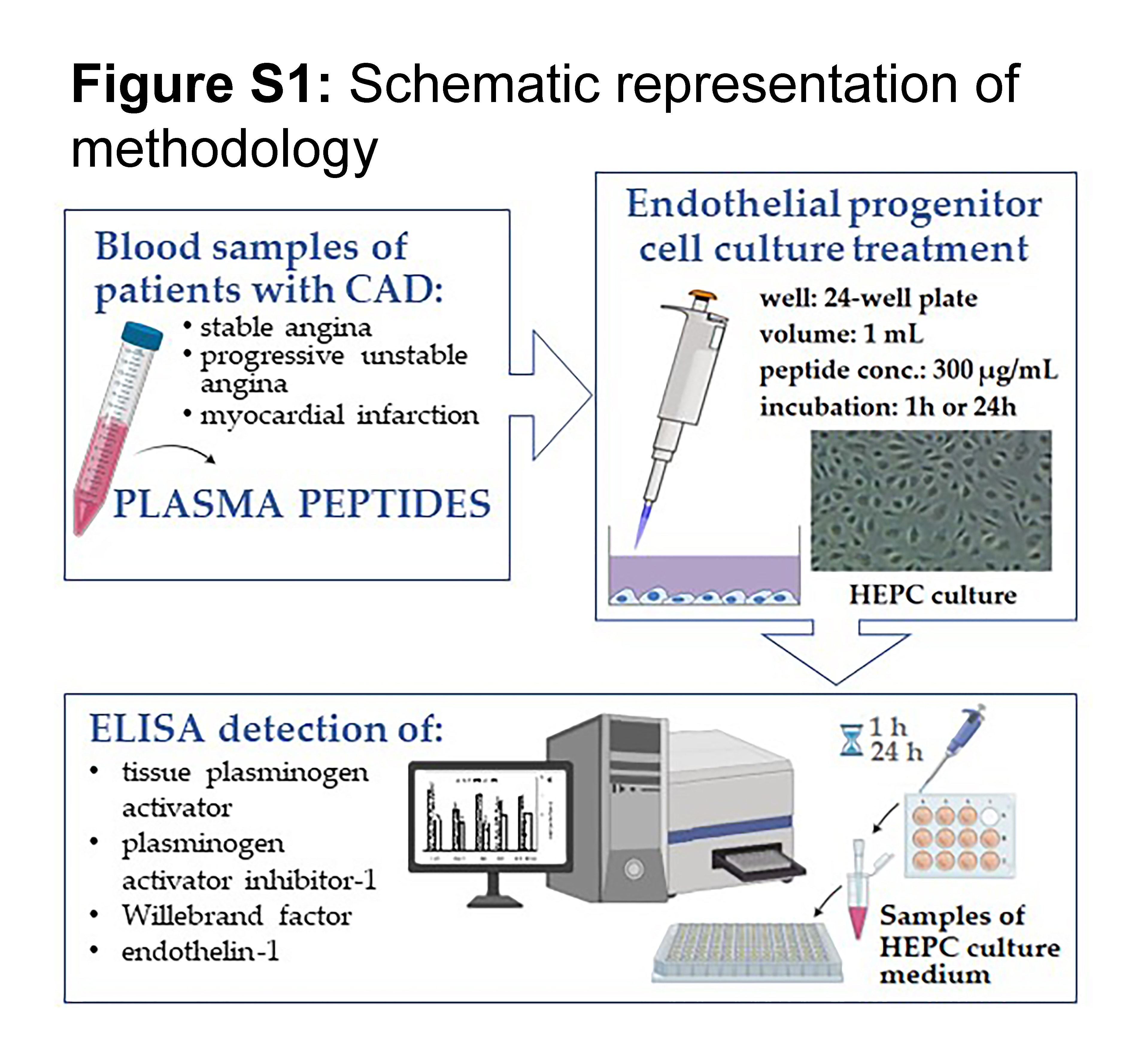

Supplement: Supplementary file 1 [file medicina-59-00238-s001.zip › medicina-2068909-SI.png]
